# Paternal characteristics associated with low birth weight among singleton births: a hospital-based birth cohort study in northern Tanzania

**DOI:** 10.11604/pamj.2021.40.179.30328

**Published:** 2021-11-24

**Authors:** Romani Roman Sabas, Noreen Nickson Nyange, Andrea Malimi Mayala, Michael Johnson Mahande, Caroline Amour, Innocent Baltazar Mboya

**Affiliations:** 1 Community Health Department, Institute of Public Health, Kilimanjaro Christian Medical University College, Moshi, Tanzania,; 2 Department of Epidemiology and Biostatistics, Institute of Public Health, Kilimanjaro Christian Medical University College, Moshi, Tanzania,; 3 School of Mathematics, Statistics and Computer Science, University of KwaZulu-Natal, Pietermaritzburg, South Africa

**Keywords:** Paternal age, paternal education, paternal occupation, maternal characteristics, Tanzania

## Abstract

**Introduction:**

low birth weight (LBW) remains a devastating adverse pregnancy outcome in low and middle income countries (LMICs). There is evidence showing that maternal demographic and pregnancy-related characteristics are associated with LBW. Little attention is given to paternal characteristics, which may be associated with a higher risk of LBW. This study aimed to assess the effect of paternal characteristics on LBW among singleton deliveries at Kilimanjaro Christian Medical Centre (KCMC) zonal referral hospital in Kilimanjaro region, northern Tanzania.

**Methods:**

this was a secondary analysis of a hospital-based cohort study from maternally-linked medical birth registry data at KCMC between 2000 and 2018. A total of 47,035 singleton deliveries were included in this study. Data analysis was performed using statistical package for social sciences (SPSS), version 20 (IBM Corp., Armonk, NY). Relative risk and corresponding 95% confidence intervals (CI) were used to determine association between LBW and paternal characteristics using log-binomial regression models, with robust standard errors to account for clustering of deliveries within mothers.

**Results:**

the proportion of LBW during the study period was 9.6%. After adjusting for maternal characteristics, higher risk of LBW was among fathers with low education level (RR=1.72, 95% CI: 1.22, 2.41, p=0.002), aged ≤24 years old (RR=1.37, 95% CI: 1.21, 1.55), and those unemployed (RR= 1.11, 95% CI: 1.01, 1.21). Lower risk of LBW was among fathers aged ≥40 years (RR=0.97, 95% CI: 0.88, 1.08), but this association was not statistically significant.

**Conclusion:**

the study confirmed paternal young age (≤24 years old), paternal low education level and unemployment as predictors for LBW. Current evidence on the effect of paternal characteristics on LBW might suggest that programs and policies should target their engagement as a key strategy for improving birth outcomes during the perinatal period. Future studies should assess how paternal factors are associated with the risk of adverse birth outcomes.

## Introduction

Low birth weight (LBW) remains to be devastating adverse pregnancy outcomes in low- and middle-income countries (LMICs) [1]. According to the World Health Organization (WHO) study of 2019 on the global burden of diseases, LBW and other perinatal causes are a leading cause of death and disability among children below five years of age [2]. More than 20 million infants worldwide, representing 15.5% of all births are born with LBW [3]. Of the 20 million LBW babies more than half were born in Asia while Africa was home to about one-quarter of all LBW newborns, with the majority born in Eastern and Western Africa [3]. The prevalence of LBW in Tanzania has dramatically reduced from 14% in 1991-1992 to 7.1% in 2010 and 7.0% in 2015-16 [4]. Low birth weight is associated with a higher risk of morbidity, mortality and long-term life health consequences including a higher risk of stunted growth, lower IQ, and adult-onset chronic conditions such as obesity and diabetes [2]. Low birth weight is attributed to multiple economic, maternal, psychosocial, and health behavior factors and represents a large burden for women, families, communities, and health-care providers [5]. Maternal characteristics such as advanced maternal age [6,7] preeclampsia, obesity, smoking during pregnancy, anemia [7,8] and previous history of LBW [7]. HIV positive status of the mother [9] maternal deprived socio-economic conditions [10] and maternal infections such as malaria and syphilis are known factors that increase the risk of LBW. Studies in the developed countries have shown that paternal characteristics such as advanced paternal age [11,12] low education level [12] unemployment [10,13] preconception smoking [8] genetic characteristics [12,14,15] depression, low social support to women [16] and exposure to environmental substances such as pesticides/ herbicides/ professional paints [17] are associated with LBW. The role of paternal characteristics in determining the risk of LBW in LMICs has received less attention than maternal factors.

Paternal and maternal unemployment has been associated with LBW in Nigeria [10] while higher paternal age (>45) and low paternal education level has been associated with the risk of LBW in Tanzania and Pakistan [13,18,19]. Most of these studies, however, are limited to a single risk factor, and have inadequate control for the effects of maternal characteristics [10,19]. Until now, preconception health counseling which has been shown to improve maternal health status and in some cases, have been demonstrated to be effective in limiting the incidence of adverse pregnancy outcomes such as LBW has mainly targeted women [17]. Prospective fathers should also be a target in preconception interventions [20]. Despite efforts to promote male involvement in maternal and child health services in Tanzania, the proportion of male participation at antenatal care (ANC) services is still low (30%) [21,22]. Partner support during antenatal care visits is an important and potentially modifiable intervention to improve pregnancy outcomes. Using data from a maternally linked birth cohort data, we aimed to assess the effect of paternal characteristics on LBW among singleton births at KCMC zonal referral hospital in Kilimanjaro region, northern Tanzania. Findings from this study may provide essential information on the influence of paternal characteristics on LBW. The study will provide evidence for the need of programs and policies to target male engagement in pregnancy. The objective of this study is to determine the association between paternal characteristics and LBW among singleton births at KCMC zonal referral hospital, northern Tanzania. Our specific aims were to; 1) determine the proportion of LBW by paternal characteristics among singleton deliveries at KCMC referral hospital, northern Tanzania, year 2000-2018; and 2) determine paternal characteristics associated with low birth weight among singleton deliveries within the same study period. We hypothesized that paternal characteristics are not associated with LBW among singleton deliveries at KCMC referral hospital, Kilimanjaro region, northern Tanzania (Null hypothesis, H0). We also hypothesized that paternal characteristics are associated with LBW among singleton deliveries at KCMC referral hospital, Kilimanjaro region, northern Tanzania and childcare as a key strategy for improving birth outcomes during the perinatal period. (Alternative hypothesis, H1).

## Methods

**Figure 1:**
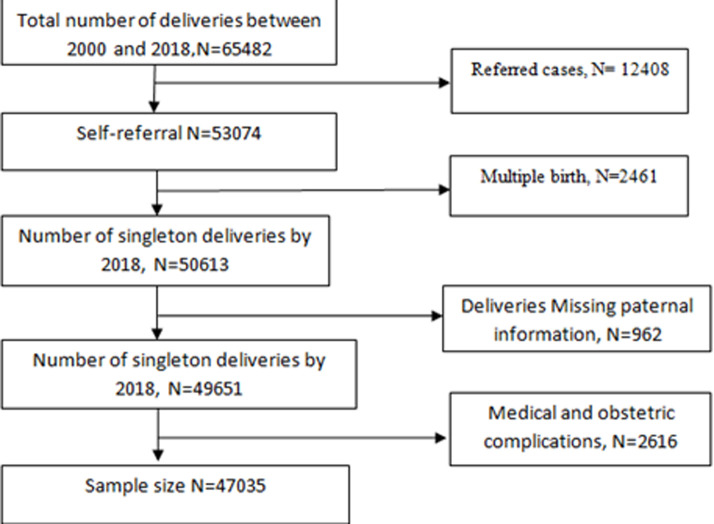
flow-chart showing the number of deliveries analyzed

**Study design, setting, and population:** we conducted a retrospective cohort study utilizing data from the birth registry at KCMC referral hospital, situated in the Moshi Municipality of Kilimanjaro region, Northern Tanzania. KCMC is one of the four zonal referral hospitals in Tanzania, and serves over 15 million people, including women from the local communities (who may come to deliver on their own accord) and referred cases from neighboring regions. An average of 4000 births occur at KCMC each year [23]. The study population was 65,482 deliveries recorded at KCMC medical birth registry from 2000 to 2018. We included 47,035 deliveries with complete records between 2000 and 2018. We excluded women with multiple deliveries (N=3461), those with missing data on both the outcomes and explanatory variables (1578), as well as births from women referred for delivery (13408). We also excluded women with medical conditions during pregnancy such as diabetes (107), hypertension (124), anemia (676), as well as those who experienced obstetric complications such as preeclampsia/eclampsia (1709), abruption placenta and placenta previa to avoid over-representation of high-risk pregnancies [7,24]. We therefore, analyzed data for 47,035 deliveries (Figure 1).

**Data source and collection methods:** the data collection methods for the KCMC medical birth registry are well described elsewhere [23,25]. In brief, data are collected about the mother and father: education, occupation and living conditions, mother's health before and during present pregnancy, expected date of delivery, smoking and drinking (alcohol) habits, use of drugs, plus HIV and syphilis status (if known). This is followed by particulars on the delivery whereby a trained midwives collect data using a standardized questionnaire (within 24 hours after delivery or later if a mother had recovered from complications). Additional data are also abstracted from the antenatal care (ANC) cards and the mother's hospital medical records. Unique identification numbers are used to identify and link women with their subsequent deliveries in the obstetrics and gynecology department at KCMC hospital. The collected data are later entered into a computerized database system at the medical birth registry located in the hospital premise´s reproductive health unit [7,18,26].

**Study variables:** the dependent variable in this study was LBW. LBW was defined as a birth weight of less than 2500g regardless of gestational age, [2,7] and was categorized into a binary variable as normal (weight at birth of ≥2500 grams) and LBW (weight at birth of <2500 grams). The primary independent/exposure variables in this study were paternal characteristics, including age in complete years at the time of childbirth (15-19, 20-24, 25-34, 35-39 and 40+ years). We used the 25-34 years group as the reference category because this is the most common age range for having children among fathers in this setting and the one showing the lowest prevalence of LBW [4,11]. Other paternal background characteristics include the area of residence (rural and urban), level of education (none, primary and secondary/higher), and occupation (unemployed and employed). We considered maternal characteristics as potential confounders; hence they were adjusted in the analysis. These included maternal age, education, occupation and the area of residence categorized in similar manner as paternal characteristics, number of antenatal care visits (<4, ≥4), marital status (married, not married).

**Statistical analysis:** we performed data cleaning and analysis using the Statistical Package for Social Sciences (SPSS version 20) [27]. Numeric variables were summarized using mean/median and corresponding measures of dispersion, i.e. standard deviation/ interquartile range. Categorical variables were summarized using frequency or proportions from the total. Relative risk (RR) and the corresponding 95% confidence intervals (CI) was used to determine the association between LBW and paternal characteristic using log-binomial regression model, with robust standard errors adjusted for clustering of observations within mothers. The multivariable regression model was adjusted for maternal characteristics. Variables with a p-value of <0.05 were considered significantly associated with LBW.

## Results

**Social demographics of the participants:** the mean paternal age among 47,035 deliveries in this study was 33 (SD=7.0) years. Majority (54%) were aged between 25-34 years. More than half (60%) had secondary or higher education level, two-third (71%) were unemployed. The majority (55%) of the mothers were aged between 25-34 years, with their mean age of 28 (SD=6.0) years. In all, 86.3% were married and 50.1% had secondary/higher education, 76% were unemployed, and 34% were residing in rural areas with only 55% attended more than 4 ANC visits (Table 1).

**Table 1:** social demographics and proportion of LBW by paternal and maternal characteristics among singleton deliveries (N=47,035)

Variables	N (%)	Normal n (%)	LBW n (%)	P-value
**Paternal Characteristics**				
**Age groups (years**)				<0.001
15-19	213 (0.5)	187 (87.8)	26 (12.2)	
20-24	3481 (7.3)	3040 (87.3)	441 (12.7)	
25-34	25362 (54)	23042 (90.9)	2320 (9.1)	
35-39	10026 (21.4)	9135 (91.1)	891 (8.9)	
≥40	7953 (16.9)	7126 (89.6)	827 (10.4)	
**Education level**				<0.001
None	257 (0.5)	215 (83.7)	42 (16.3)	
Primary	18682 (39.9)	16560 (88.6)	2122 (11.4)	
Secondary/higher	28096 (60.0)	25755 (91.7)	2341 (8.3)	
**Occupation**				<0.001
Unemployed	33414 (71.0)	29954 (89.6)	3460 (10.4)	
Employed	13621 (29.0)	12576 (92.3)	1045 (7.7)	
**Maternal characteristics**				
**Age groups (years)**				<0.001
15-19	2850 (6.0)	2538 (89.1)	312 (10.9)	
20-24	10818 (23.0)	9758 (90.2)	1060 (9.8)	
25-34	25733 (54.8)	23432 (91.1)	2301 (8.9)	
35-39	5933 (12.6)	5310 (89.5)	623 (10.5)	
≥40	1627 (3.5)	1433 (88.1)	194 (11.9)	
**Occupation**				0.001
Unemployed	35948 (76.4)	32242 (89.7)	3706 (10.3)	
Employed	11087 (23.6)	10288 (92.8)	799 (7.2)	
**Education level**				0.001
Primary	23508 (49.9)	20955 (89.1)	2553 (10.9)	
Secondary/higher	23527 (50.1)	21575 (91.7)	1952 (8.3)	
**Marital Status**				<0.001
Not married*	6466 (13.7)	5722 (88.5)	744 (11.5)	
Married	40569 (86.3)	36808 (90.7)	3761 (9.3)	
**Area of residence**				<0.001
Rural	15999 (34.0)	14238 (89.0)	1761 (11.0)	
Urban	31036 (66.0)	28292 (91.2)	2744 (8.8)	
**Number of ANC visits**				<0.001
<4	25388 (55)	22092 (87.0)	3296 (13.0)	
≥4	20996 (45)	19921 (95.0)	1075 (5.1)	
**Overall**	47035	42530 (90.4)	4505 (9.6)	

Notes; *means living alone without a partner

**The proportion of LBW by paternal characteristics among singleton deliveries:** the overall proportion of LBW among singleton deliveries in this cohort was 9.6%. There was a significant association between LBW and paternal age, education level and occupation. The proportion of LBW was high (13%) among fathers aged less than 24 years, compared to fathers aged 25-34 (9.1%). LBW was also higher among fathers with no or primary education (16.3%), and those unemployed (10.4%) (Table 1).

**Paternal characteristics associated with:** there was a significant association between LBW and paternal age, education level, and occupation in the unadjusted/crude analysis. The risk of LBW was high among fathers aged ≤24 years old (RR=1.44, 95%CI 1.29, 1.60), and ≥40 years (RR=1.15, 95%CI 1.06-1.25) compared to those aged 25-34, with low education (RR=2.15, 95%CI 1.54-2.90), the unemployed (RR=1.40, 95%CI 1.30, 1.50) (Table 2). However, in the adjusted analysis (adjusted for maternal characteristics), the risk of LBW continued to be relatively higher among fathers with low education level (RR=1.72, 95%CI 1.22, 2.41, p=0.002), aged ≤24 years old (RR=1.37, 95%CI 1.21, 1.55), and those unemployed (RR=1.10, 95%CI 1.01, 1.19). There was lower risk of LBW among fathers aged ≥40 years (RR=0.97, 95%CI 0.88, 1.08), but this association was not statistically significant (Table 2).

**Table 2:** the association between LBW and paternal characteristics among singleton deliveries (N=47035)

Variables	cRR* (95%CI)	P-value	aRR** (95%CI)	P-value
**Age groups (years**)				
15-19	1.40 (0.94-2.09)	0.02	1.31(0.85-2.02)	0.20
20-24	1.44 (1.29-1.60)	0.001	1.37 (1.21-1.55)	0.001
35-39	0.97 (0.89-1.05)	0.44	0.92 (0.84-1.003)	0.06
≥40	1.15 (1.06-1.25)	0.001	0.97 (0.88-1.08)	0.69
25-34	1.00		1.00	
**Education level**				
None	2.15 (1.54-2.90)	<0.001	1.72 (1.22-2.41)	0.002
Primary	1.41 (1.33-1.50)	<0.001	1.19 (1.10-1.28)	<0.001
Secondary/higher	1.00		1.00	
**Occupation**				
Unemployed	1.40 (1.30-1.50)	0.003	1.10 (1.01-1.19)	0.040
Employed	1.00		1.00	

**Notes: cRR;** crude risk ratio **aRR;** adjusted risk ratio, adjusted for maternal age, occupation, education, area of residence, marital status and number of ANC visits

## Discussion

The study aimed to determine the association between paternal background characteristics and LBW among singleton deliveries at KCMC referral hospital, northern Tanzania. The proportion of LBW in all recorded deliveries from 2000-2018 was 9.6%. Independent of maternal characteristics, low paternal education, unemployed, and low paternal age (≤24 years) increased the risk of LBW. The proportion of LBW in this study is higher than the national estimate (7%) [4]. The fact that the national estimate comes from the Tanzania Demographic and Health Survey, which is population-based, compared to this study that uses data from a tertiary care hospital, might explain these differences. Our findings are somehow consistent to most of the hospital-based studies in sub-Saharan Africa (9.47%) [28] with prevalence ranging from 8.9% in eastern Africa to 10.3% in southern Africa, and the lowest prevalence reported 6.3% in Guinea, 7.3% Nigeria among the western African countries [10,28]. The estimate in this study is lower than 16%, globally [2] which might be due to the differences in the study population, sample size and the social-economic status across the study populations. It is worth noting that LBW proportions might be higher than the reported because the data on LBW remains limited to the most developing countries, including Tanzania, whereas half of deliveries occur at home [3,29]. There was an association between low paternal education level and unemployment with a high risk of LBW, similar to other studies [12]. The association between low paternal education level may be explained by the low level of awareness on the available health services and decrease accessibility and utilization of available health information and technology as a consequence of low education [13,15]. Higher paternal education level could improve their social-economic status, increase their level of awareness on maternal health-related issues and subsequently lower the risks of LBW [10]. Hence, obtaining information on paternal education levels may improve our ability to identify groups at highest risk for LBW. This may allow the development and implementation of the targeted interventions to reduce the risk of LBW among other adverse pregnancy outcomes [10]. Strategies to improve the availability, accessibility, and affordability of maternal health services should be focused on fathers with low education levels.

Unemployment significantly increased the risk of LBW, which could be associated with poor socio-economic status.[10,13] Poor socio-economic status has been linked to inadequate access to quality reproductive health care services, poor household nutrition, and hence increased risks of adverse pregnancy outcomes, including LBW [13]. Low paternal age was associated with a higher risk of LBW, as also reported in Finland and Canada [11]. On the contrary, a study done in the USA revealed that paternal age of more than 35 years increased the risk of LBW [30]. However, this study included only maternal age as a confounder, excluding other important maternal background characteristics and complications which were included in our study. Paternal genetic changes due to age might play a role in the risk of LBW associated with abnormal placenta formation [15]. The influence of younger paternal age on LBW is, however, not clear [12]. Therefore, more studies should further investigate how paternal characteristics influence LBW, among other adverse pregnancy outcomes. The observed association between paternal characteristics and LBW emphasizes the need for male involvement in maternal and child health services. This allows for early identification and management of high-risk pregnancies, accounting for the contribution of paternal-related factors. Research in low and middle-income countries have reported low male participation in maternal and child health services [22]. Also, male involvement in maternal and child health services in Tanzania is still a challenge. For instance, only 30% of men in Tanzania participate at ANC [21]. Health care providers and program implementers should take appropriate action to advocate and encourage men´s involvement in ANC. Health education and services provided during ANC have the potential to reduce pregnancy and delivery complications, including LBW, among other birth outcomes [22].

**Strengths and limitations:** the large hospital-based birth registry data recorded from 2000-2018 in the KCMC zonal referral hospital enabled us to analyze and establish the association between paternal characteristics and LBW controlling for potential maternal confounders. This study is probably the first, to the best of our knowledge, to report the association between paternal characteristics and LBW in Tanzania and sub-Saharan Africa. As with other epidemiological studies, some key variables, for instance lifestyle factors of the fathers, such as smoking and alcohol use, were not measured and included in the birth registry, which has been associated with LBW in other studies [8]. The study involved deliveries recorded in a tertiary hospital, which might not reflect the true picture in the general Tanzanian population.

## Conclusion

The study confirmed paternal age, education level and occupation as predictors for LBW. Current evidence on the effect of paternal characteristics on LBW might suggest that programs and policies should target their engagement as a key strategy for improving birth outcomes during the perinatal period. Future studies should assess how paternal factors are associated with the risk of adverse birth outcomes.

### What is known about this topic

- Maternal characteristics are known factors that increase the risk of LBW;
- Preconception health counseling which has been shown to improve maternal health status and, in some cases, have been demonstrated to be effective in limiting the incidence of adverse pregnancy outcomes such as LBW has mainly targeted women.

### What this study adds

- This study confirmed paternal young age (≤24 years old), paternal low education level and unemployment as predictors for LBW;
- Programs and policies should target paternal engagement as a key strategy for improving birth outcomes during the perinatal period.
